# Preliminary Evaluation of In Vitro Bacteriostatic and Bactericidal Effect of Salt on *Leptospira* spp.

**DOI:** 10.3390/vetsci7040154

**Published:** 2020-10-13

**Authors:** Giovanni Cilia, Filippo Fratini, Elena della Buona, Fabrizio Bertelloni

**Affiliations:** Department of Veterinary Sciences, University of Pisa, Viale delle Piagge 2, 56124 Pisa, Italy; Giovanni.cilia@vet.unipi.it (G.C.); elenadellabuona@hotmail.it (E.d.B.); fabrizio.bertelloni@unipi.it (F.B.)

**Keywords:** *Leptospira*, MIC, microdilutions method, salt susceptibility, leptospirosis, environmental resistance, halotolerance, seawater

## Abstract

Environmental resistance is an important factor for understanding the epidemiology of leptospirosis. Recently, new *Leptospira* hosts were identified, including also marine mammals. Moreover, halotolerant *Leptospira* strain, isolated from the environment and animals, highlighted the capability of this microorganism to persist in the seawater. The aim of this research was to investigate the bacteriostatic and bactericidal effect of salt on *Leptospira* strains belonging to 16 different serovars. The minimum inhibitory concentration (MIC) and minimum bactericidal concentration (MBC) values were verified through the microdilutions method starting from a 20% sodium chloride concentration. MIC values obtained were between 0.3125% and 10% of salt, while MBC values between 0.625% and >20%. Icterohaemorrhagiae (MIC: 0.3125%; MBC: 0.625%) resulted the most inhibited serovar, while the most resistant was Tarassovi (MIC: 10%; MBC: >20%). Interestingly, trends were reported for Pomona (MIC: 1.25%; MBC: >20%) and Bratislava (MIC: 0.625%; MBC: 20%), highlighting low MIC values but high MBC values. This is the first investigation aimed at the *in vitro* effect of salt on the growth of *Leptospira* spp. reference strains.

## 1. Introduction

Leptospirosis is a re-emerging zoonosis caused by pathogenic *Leptospira*, bacteria belonging to Spirochetales order [1,2]. Leptospirosis is worldwide diffused and represents an important public health problem, the bacterium causes from mild to severe infection in both humans and animals [2]. Humans, domestic and wild animals represent *Leptospira* maintenance or accidental hosts [1,3,4,5,6]. Recently, some *Leptospira* serovars seem to be able to infect atypical hosts [7,8]. Indeed, new hosts were identified among domestic and wild animals [9,10], including also marine mammals [11,12,13,14,15]. Among marine mammals, *Leptospira* infection was evidenced in pinnipeds, such as California sea lions (*Zalophus californianus*) [12,16,17,18,19,20], Northern elephant seals (*Mirounga angustirostiris*) [21], Chilean South American sea lions (*Otaria byronia*) [22], Northern fur seals (*Callorhinus ursinus*) [23] and harbor seals (*Phoca vitulina*) [24,25]. Moreover, signs amenable to leptospirosis and serological positivity were reported in Peruvian Amazon manatees (*Trichechus inunguis*) [26] and bottlenose dolphin (*Tursiops truncatus*) [15], while a new *Leptospira* strain named Manara was isolated from Southern right whale (*Eubalena australis*) [11]. The epidemiology of leptospirosis is strictly connected to environmental factors, including presence of water and its osmolarity; this medium represents one of the most important transmission routes making possible the *Leptospira* spread in the ecosystem [27,28].

Although *Leptospira* growth and survival were considered to be incompatible with saline water [29,30,31,32], *Leptospira biflexa* strain Muggia and strain Ancona Porto were isolated from seawater near Trieste (Italy) and near Ancona, respectively [33,34,35], and two unidentified *Leptospira* strains, isolated in Philippines from soil samples after a Super typhoon, were able to grow in artificial seawater for four and three days, respectively [36]. Additionally, the *Leptospira* strain Manara was able to grow in a medium with 10% of salt for more than two weeks [11].

Considering the new evidence about *Leptospira* epidemiology and the missing of accurate data on the *in vitro* resistance of this bacterium to salt, the aim of this research was to investigate, using the microdilution method, the bacteriostatic and bactericidal effect of salt on *Leptospira* strains belonging to 16 different serovars.

## 2. Materials and Methods

### 2.1. Leptospira spp. Strains

The reference *Leptospira* strains employed in this investigation were the same as previously used, reported in Table 1.

Each strain was maintained in pure culture in liquid Ellinghausen–McCullough–Johnson–Harris (EMJH) medium (Difco, Detroit, MI, USA), subcultured and weekly checked for purity and viability.

### 2.2. Minimal Inhibitory Concentration (MIC) and Minimal Bactericidal Concentration (MBC)

Sterile NaCl was added to EMJH medium in order to reach a stock solution with 21.05% of salt; this allowed to start to test a concentration of 20% of NaCl, considering the inoculum addition, as reported below. The minimum inhibitory concentration (MIC) determination was performed by the broth microdilution method, as previously reported [37,38]. *Leptospira* cultures were quantified with spectrophotometry using optical density at 420 nm (Multiskan™ FC Microplate Photometer; Thermo Fisher Scientific, Haverhill, MA, USA) to reach a concentration which corresponds approximately to 1.5 × 10^8^ cfu/mL: considering that an OD ranging between 0.052 to 0.1 corresponds to approximately 2–3 × 10^8^ leptospires per mL, cultures were diluted in EMJH to reach this OD, using EMJH as blank; after, standardized culture was diluted 1:2 in EMJH to reach the desired inoculum concentration [39,40]. The procedure to assess the MIC and minimum bactericidal concentration (MBC) is the same previously reported [37]. In detail, in each well of the 96-well plates, two-fold serial dilutions were performed in EMJH/salt medium, ranging from a 20% to 0.03% salt concentration. The final volume in each well was 100 μL, including 5 μL of standardized strain. Two microplates were prepared for each strain: one to determine the MIC and the other for the MBC. They were incubated for 3 days at 30 °C, then 20 μL of resazurin sodium salt (Alfa Aesar, Thermo Fisher Scientific, Haverhill, MA, USA), diluted 1:30 in EMJH medium (10% stock solution was used reaching a final concentration of 0.33%), was added to each well in the plate used for MIC. The plates were incubated at 30 °C until 4 weeks. Change in color from blue to pink indicated *Leptospira* growth: resazurin is a cell growth indicator dye that turns from dark blue to bright pink when growing organisms are present. The MIC value was reported as the lowest concentration able to prevent a color change.

MBC were determined by plating 10 μL from each well (starting from the MIC value to higher salt concentrations) in 1.5% EMJH agar. Plates were incubated at 30 °C for 10 days. The MBC value was reported as the lowest concentration able to kill 99.9% of the inoculated bacteria. Additionally, for each strain a positive control (EMJH without salt) and a negative control (only EMJH) were employed.

All tests were performed in triplicate and the results were expressed as median.

## 3. Results

Table 2 shows the MIC and MBC values obtained for the different strains of *Leptospira* spp.

MIC values resulted between 0.3125% and 10% of salt, while MBC values between 0.625% and >20%. Icterohaemorrhagiae was the most inhibited serovar with MIC and MBC of 0.3125% and 0.625%, respectively. Moreover, the most resistant serovar was Tarassovi, with values of 10% for MIC and >20% for MBC.

Interestingly trends were reported for serovar Pomona (1.25% for MIC and >20% for MBC) and Bratislava (0.625% for MIC and 20% for MBC), highlighting low MIC values but high MBC values. Additionally, serovar Zanoni did not survive all assays, even at low salt concentration (0.03%).

## 4. Discussion

To the best of the authors’ knowledge, this is the first investigation showing the *in vitro* effect of salt against *Leptospira* spp. reference strains. Results showed that some *Leptospira* strains seem to be halotolerant and could be able to grow up in saline water. To the best of the authors’ knowledge, no studies focused on the effect on *Leptospira*, excluding isolation from marine mammals. Few previous studies have evaluated the *Leptospira* halotolerance or their growth in the marine environment. In Italy, the isolation of *Leptospira biflexa* strain Muggia was performed from marine water sampled in the Adriatic Sea, near Trieste [33,34] and *Leptospira interrogans* serovar Pomona was isolated from a bottlenose dolphin (*Tursiops truncatus*) stranded in Sardinian coast [15]. Moreover, the growth capability in a saline environment was verified for two *Leptospira* strains isolated from the soil in the Philippines following the super Typhoon Haiyan (Yolanda). Both isolates were able to grow in both sea water and artificial saline environment, surviving for 4 and 3 days, respectively [36]. Finally, the first *Leptopsira* strain named Manara, isolated in Argentina from a *Eubalena australis* was alive and viable for more than 12 days in media with different salt concentrations up to 10% [11].

In this investigation, almost all of tested *Leptospira* strains were inhibited and killed by a salt concentration much lower than marine salinity (3.5%), but some of them resulted resistant also at high salt concentration. The bacteriostatic values reported for Pomona and Bratislava showed that these serovars were inhibited by salt concentration lower than one present in the sea (3.5%). However, considering MBC values, it could be hypothesized that these serovars could survive in saline water for some days without multiplication, allowing the possible infection of marine mammals [11,13,15,41,42].

It is also noteworthy that Pomona is the most commonly detected serovar in a lot of marine mammals, from pinnipeds to dolphins. Several leptospirosis outbreaks in California sea lions have been caused by *Leptospira interrogans* serovar Pomona, that seem to be enzootic and able to cause abortion and renal diseases [12,16,17,18,19,20], also persisting in the renal tubules of bottlenose dolphins [15]. The high prevalence of Pomona in marine mammals could be related to high, intrinsic, and remarkable virulence of this serovar, able to infect and cause disease to different kinds of hosts; however, it could probably be linked also to its resistance to different environmental conditions, as well as high salt concentration, that could make this serovar highly adaptable to different kinds of ecosystems and, in this way, increasing the opportunity to come into contact with new potential susceptible hosts.

As for Pomona, the capability to live in salt solution of serovar Bratislava could be potentially linked to other features for environmental resistance, also justified to the re-emergence and high diffusion of this serovars in several areas [3,43,44,45,46].

On the other hand, the high salt inhibition of serovars Icterohaemorrhagiae and Copenhageni, both belonging to serogroup Icterohaemorrhagiae, could be related to their epidemiologic features. Indeed, rats and mice are recognized as *reservoirs* of these serovars, and the rapid spread of the disease is favored by the high number of animals, the close contact between specimens, and their high level of prolificacy. These features allow the constant presence of new susceptible animals [47,48]. Therefore, in this case, the low salt resistance of these serovars could negatively affect their environmental resistance and, nevertheless, the great availability of susceptible hosts could guarantee the spread of the infection and the maintenance of the serovars, even in cases of low environmental resistance.

Finally, regarding serovar Tarassovi, although the obtained data suggested a high resistance of the serovar Tarassovi to salt, which could favor a high environmental resistance, the prevalence of the infection due to *L*. Tarassovi is decreasing as emerged by recent investigations [3,49,50]. However, the reported high salt resistance, probably connected to a good environmental resistance, could be strictly connected to its epidemiology. This serovar, reported almost only in swine [51,52,53], seems to have not completely disappeared, but it occurs sporadically with low prevalence; perhaps it could remain in the environment for a long time even in the absence of a *reservoir* [30].

## 5. Conclusions

In conclusion, the results of this investigation highlighted various grades of halotolerance among *Leptospira* strains. Although most of the serovars showed low salt resistance, some strains resulted tolerant to high salt concentration, considering MIC, MBC, or both. Employed strains are not recent isolates from sea mammals or marine environments, but they are reference *Leptospira* strains; for this reason, obtained results seem to be suggestive of an intrinsic resistance and not the consequence of a new adaptation of this bacterium to different ecosystems. Salt resistance could partially explain some differences in epidemiology of different serovars and could be useful to identify new potential risk factors related to leptospirosis. However, to better understand the mechanisms of action underlying this phenomenon, further research will have to be carried out and other serovars must be tested as well as reference and filed strains isolated from different sources.

## Figures and Tables

**Table 1:** Panel of 16 investigated *Leptospira* spp. strains used in this investigation.

*Leptospira* Species	Serogroup	Serovar	Strain
*Leptospira interrogans*	Icterohaemorrhagiae	Icterohaemorrhagiae	Bianchi
*Leptospira interrogans*	Canicola	Canicola	Alarik
*Leptospira interrogans*	Pomona	Pomona	Mezzano
*Leptospira borgpetersenii*	Tarassovi	Tarassovi	Mitis Johnson
*Leptospira kirschneri*	Grippotyphosa	Grippotyphosa	Moska V
*Leptospira interrogans*	Australis	Bratislava	Riccio 2
*Leptospira borgpetersenii*	Ballum	Ballum	Mus 127
*Leptospira interrogans*	Sejroe	Hardjo	Hardjoprajitno
*Leptospira interrogans*	Icterohaemorrhagiae	Copenhageni	Wijmberg
*Leptospira interrogans*	Bataviae	Bataviae	Pavia
*Leptospira interrogans*	Pyrogenes	Zanoni	Zanoni
*Leptospira borgpetersenni*	Javanica	Poi	Poi
*Leptospira interrogans*	Australis	Lora	Riccio 37
*Leptospira interrogans*	Autumnalis	Autumnalis	Akiyami
*Leptospira interrogans*	Habdomadis	Hebdomadis	Hedbomadis
*Leptospira biflexa*	Semaranga	Patoc	Patoc I

**Table 2:** Minimum inhibitory concentration (MIC) and minimum bactericidal concentration (MBC) for salt evaluated for the 16 investigated *Leptospira* spp. strains. Results are expressed as median and values reported in % of salt in Ellinghausen–McCullough–Johnson–Harris (EMJH) medium.

Serovar	Salt	
	MIC	MBC
Icterohaemorrhagiae	0.3125	0.625
Canicola	0.625	0.625
Pomona	1.25	>20
Tarassovi	10	>20
Grippotyphosa	1.25	1.25
Bratislava	0.625	20
Ballum	0.625	0.625
Hardjo	1.25	1.25
Copenhageni	0.625	1.25
Bataviae	1.25	1.25
Zanoni	-	-
Poi	1.25	1.25
Lora	1.25	1.25
Autumnalis	1.25	1.25
Hebdomadis	1.25	2.5
Patoc	1.25	1.25

-: the strain did not survive all assays.
